# Fluctuating thermal environments of shallow-water rocky reefs in the Gulf of California, Mexico

**DOI:** 10.1038/s41598-019-53730-0

**Published:** 2019-12-02

**Authors:** Grantly R. Galland, Philip A. Hastings, James J. Leichter

**Affiliations:** University of California San Diego, Scripps Institution of Oceanography, Biology Section, La Jolla, CA 92039 USA

**Keywords:** Biogeography, Marine biology

## Abstract

As part of a broad-scale study of the biogeography of rocky reefs in the Gulf of California, Mexico (GOC), we collected a continuous 1-yr temperature time series at ~5 m water depth at 16 sites spanning 5° of latitude and ~700 km along the western boundary of the basin. Throughout the region, thermal conditions were most variable in summer with fluctuations concentrated at diurnal and semi-diurnal frequencies, likely associated with solar and wind forcing and vertical water column oscillations forced by internal waves. Temperatures in winter were less variable than in summer, and minimum temperatures also differed among sites. Thermal variability integrated across the diurnal and semi-diurnal frequency bands was greatest near the Midriff Islands in the northern GOC and decreased toward the southern sites. Diurnal variability was greater than semi-diurnal variability at 13 of the 16 sites. A statistic-of-extremes analysis indicated shortest return times for cooling events in summer, and reef organisms at many of the sites may experience anomalous 2 to 5 °C cooling events multiple times per month. The significant extent of local temperature variability may play important roles in limiting species occurrences among sites across this biogeographic region.

## Introduction

There is growing recognition that changes in oceanographic conditions, on biologically relevant spatial and temporal scales, are important drivers of marine community ecology, and the term ‘ocean weather’ has been used to describe this high-frequency environmental variability^1^. Ocean weather is likely particularly important for sessile organisms and for mobile species that have very small home ranges and experience oceanographic variability as water masses move past them, rather than as they move in and out of water masses. Temperature variability is perhaps the most biologically relevant factor in ocean weather, given the ectothermic nature of most marine species with restricted movements (pelagic larvae notwithstanding). In the marine environment, regular variation in local temperature is known to impact survivability^2^; activity levels, behavior, and ‘personality’ (i.e., the differential change to behavior among individuals in a population) of marine organisms^3^; resilience to sudden atmospheric weather activity^4^; resilience to long-term climate change^5,6^; as well as expansion, contraction, or shifts to geographic ranges^7–9^. *In situ* measurement of environmental variability, particularly of temperature, is important for ecologists seeking to understand drivers of marine community composition and to project the influences of changing oceanographic climate on marine communities. These issues are especially relevant for regions near biogeographic transition, such as the boundary between tropical and temperate areas. The Gulf of California, Mexico is one such region.

**Figure 1:**
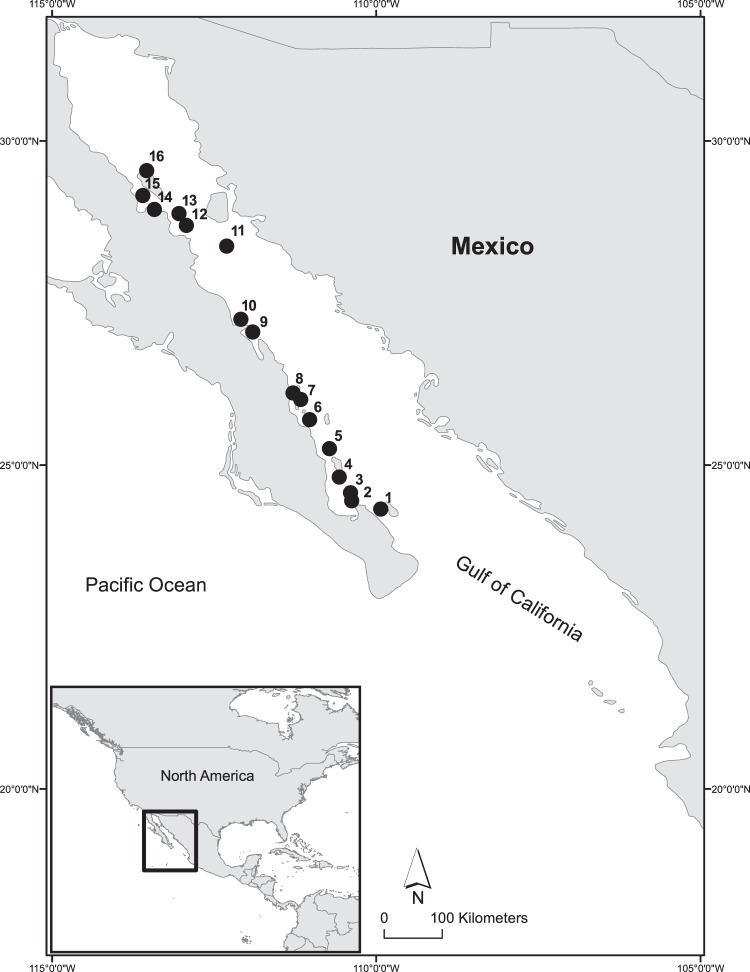
Shallow water study sites across the Gulf of California.

The Gulf of California (GOC), is a long (~1100 km) and relatively narrow (~150 km) semi-enclosed sea (Fig. 1) known for strong wind forcing, high solar insolation, and dynamic oceanographic conditions resulting from the shape, climate, and geology of the basin^10^. The North American monsoonal winds blow along the long axis of the GOC, generally from the southeast during the summer and from the northwest during the winter. This seasonal pattern produces different upwelling patterns for opposing coasts and leads to an annual reversal in the direction of the average overall GOC circulation^11^. The narrow shape of the GOC also allows for upwelled water masses at one coast to be advected to the opposite coast before undergoing significant change at the surface^12,13^. Physical conditions and variability in the GOC are also strongly influenced by internal waves associated with water column density and temperature stratification, and have been studied in the GOC since as early as the 1930s, through both *in situ* instrumentation and remote sensing. Internal waves are known to be generated near the southern portion of the GOC, through interactions with the open Pacific, and near the Midriff Islands as a result of the strong tidal forcing interacting with shallow sills between islands^14–17^.

The dynamic oceanographic conditions and prevalence of rocky reefs throughout the GOC^18^ make this an especially interesting region in which to consider the effects of the variable thermal environment on the distributions and abundance of marine organisms. The GOC is located at the intersection of tropical and temperate biogeographic provinces in the eastern Pacific^19^, and the physical conditions there vary across a wide range of temporal and spatial scales^20^. Environmental conditions in the GOC are also strongly influenced by climate-scale variability, especially the El Nino Southern Oscillation (ENSO) which exerts a range of biological responses in shallow-water marine communities^21^, and longer-term changes in climate detected at centennial scales^22^. Shallow water organisms inhabiting rocky reefs in the GOC are likely to experience variable and fluctuating physical environments, depending on location and complex interactions between local and regional upwelling, surface flow from the Pacific, solar heating, and internal waves. Multiple species of reef fishes have populations extending over much of the GOC, and are likely to experience differing degrees of oceanographic variability across their geographic ranges. Highly mobile species may also experience significant temperature variability across migratory ranges. However, the extent of local and regional temperature variation and their potential effects for both sessile and mobile benthic organisms in the GOC are not well understood.

Here we report on *in situ* temperature variability measured at shallow water rocky reefs throughout much of the GOC in 2009 and 2010 (Fig. 1). The primary goal of this study was to measure the timing and extent of temperature variability likely to be experienced by shallow-water (depths <5 m) reef organisms across a large biogeographic province. We collected and analyzed data from deployments of temperature loggers recording continuously over a full year across 16 sites, spanning ~700 km along the coast of the Baja California Peninsula along the western boundary of the GOC. We characterize the fluctuating thermal environments both seasonally, and across sites with specific attention to both diurnal and semi-diurnal frequency variability likely caused by combinations of solar heating, wind forcing, and internal waves^8^. We also calculate predicted median return times for daily minimum temperature anomalies relative to mean seasonal conditions. Recurring cooling events may be of physiological and ecological importance for rocky reef organisms, and characterizing site-specific and seasonal thermal variability across a large spatial scale is valuable for considering potential biological adaptation to the heterogeneous physical environments across the region.

## Results

### Seasonal and spatial patterns

**Figure 2:**
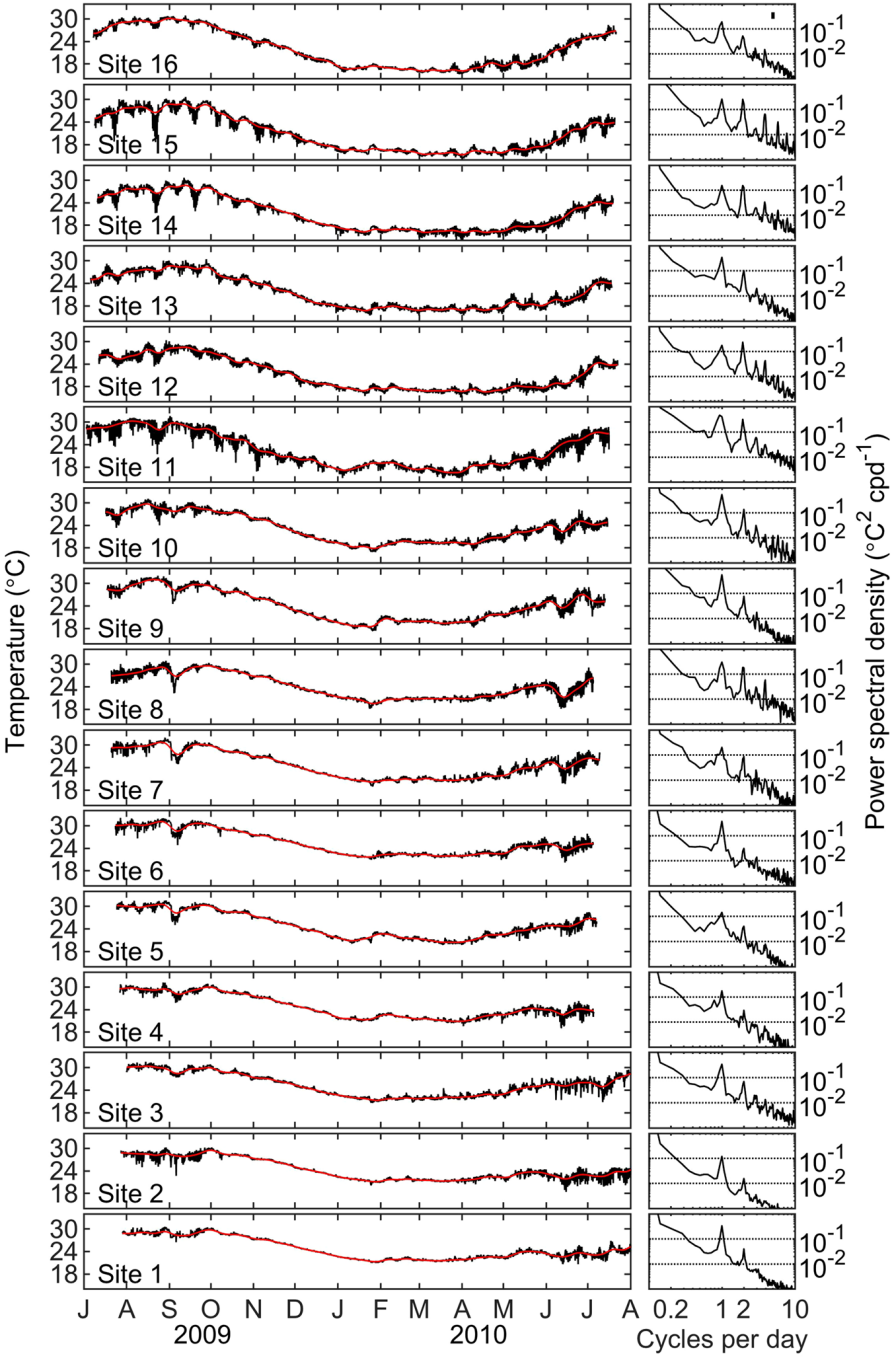
Temperature time series sampled at 20 min interval (left panel, black lines) with 29-d running mean (red) at sites across the Gulf of California. Right panels show corresponding power spectral density calculated for each site. Site numbers as in Fig. 1 and Table 1.

Summary statistics for the time series at each study site are provided in Table 1, and the full temperature time series along with 29-day centered moving means for each site are shown in Fig. 2. The minimum of the running mean temperatures ranged from 15.3 °C at Site 15 in the northern portion of the study region to 21.6 °C at Site 6. The maximum of the running mean temperatures in summer were more similar across sites, ranging from 28.4 °C at Site 4 to 31.1 °C at Site 7. Superimposed on the seasonal trends are marked patterns of higher frequency temperature variability, particularly in summer. During the warmer summer period, along with the preceding period of increase in average temperature, there was high thermal variability and departures from the mean values primarily associated with rapid decreases in temperature below the running mean conditions. By comparison, there was much less thermal variability during the period of gradual decrease in average temperature and the cooler winter months.

**Table 1:** Study site numbers, names, and location information, along with temperature summary statistics for the time series from Jul 2009 to Aug 2010.

Site #	Site Name	Lat	Lon	Mean	Min	Max	Range	Stdev
16	Puerto Refugio	29.550	113.547	22.1	15.2	30.9	15.7	4.95
15	Isla Alcatraz	29.166	113.607	20.9	14.2	30.6	16.4	4.74
14	Punta Quemada	28.950	113.425	21.1	14.1	30.7	16.5	4.49
13	Isla Partida	28.887	113.047	21.5	15.5	30.4	14.9	4.22
12	Isla Las Animas	28.705	112.934	21.2	15.1	30.3	15.3	4.17
11	Isla San Pedro Martir	28.386	112.313	22.6	15.1	31.6	16.5	4.62
10	Isla San Marcos	27.256	112.095	23.2	16.9	31.1	14.2	3.75
9	Isla Santa Inez	27.059	111.909	24.0	17.3	32.0	14.7	3.93
8	Isla Coronado	26.117	111.287	24.1	18.2	30.9	12.7	3.25
7	Isla Carmen	26.017	111.169	24.6	19.0	31.9	12.9	3.52
6	Isla Monserrate	25.710	111.033	25.3	20.2	32.1	11.9	3.21
5	Isla Santa Cruz	25.261	110.727	24.9	19.7	31.5	11.8	3.39
4	Isla San Francisquito	24.821	110.577	24.7	19.0	31.2	12.2	3.13
3	El Embudo	24.580	110.400	23.8	16.5	30.3	13.7	3.16
2	Gallina	24.458	110.383	24.2	18.1	30.6	12.5	3.16
1	Isla Cerralvo	24.326	109.937	25.2	17.7	31.6	13.8	3.46

Site numbers correspond to locations shown in map (Fig. 1) and are presented from north to south.

**Figure 3:**
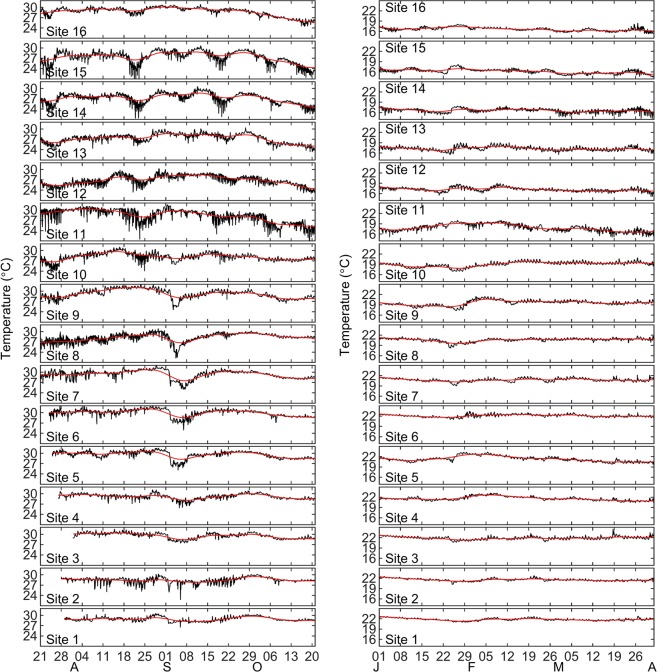
(A – Left panels) Temperature time series sampled at 20 min interval (black lines) with 29-d running mean (red lines) for summer conditions shown between 15 Jul 2009 and 15 Oct 2009, at sites across the Gulf of California; (B – Right panels) Temperature time series between 1 Jan 2010 and 1 Apr 2010.

The right-hand panels of Fig. 2 show power spectra calculated on the time series from each study site. Across all sites there were consistent peaks of temperature variability centered at the diurnal frequency (~1 cycle per day; cpd), and at the lunar semidiurnal frequency of ~1.9 cpd. Aspects of the high frequency variability and differences among sites are shown in Fig. 3A for the period July 15 to Oct 15 2019, and Fig. 3B showing Jan 1 to Apr 1 2010. The variability was markedly more pronounced in the summer than in the winter. There are also differences among sites, as well as clear similarities among sites when considered within three broad regions. The regions correspond to the northern portion in the vicinity of the Midriff Islands, the mid GOC, and the lower GOC for sites in the vicinity of La Paz. At 8 of 16 sites, more than 40% of the total annual temperature range was observed during a single day, at least once. More than 50% of the annual range was observed during a single day at two sites (sites 3 and 15).

### Time series analysis

**Figure 4:**
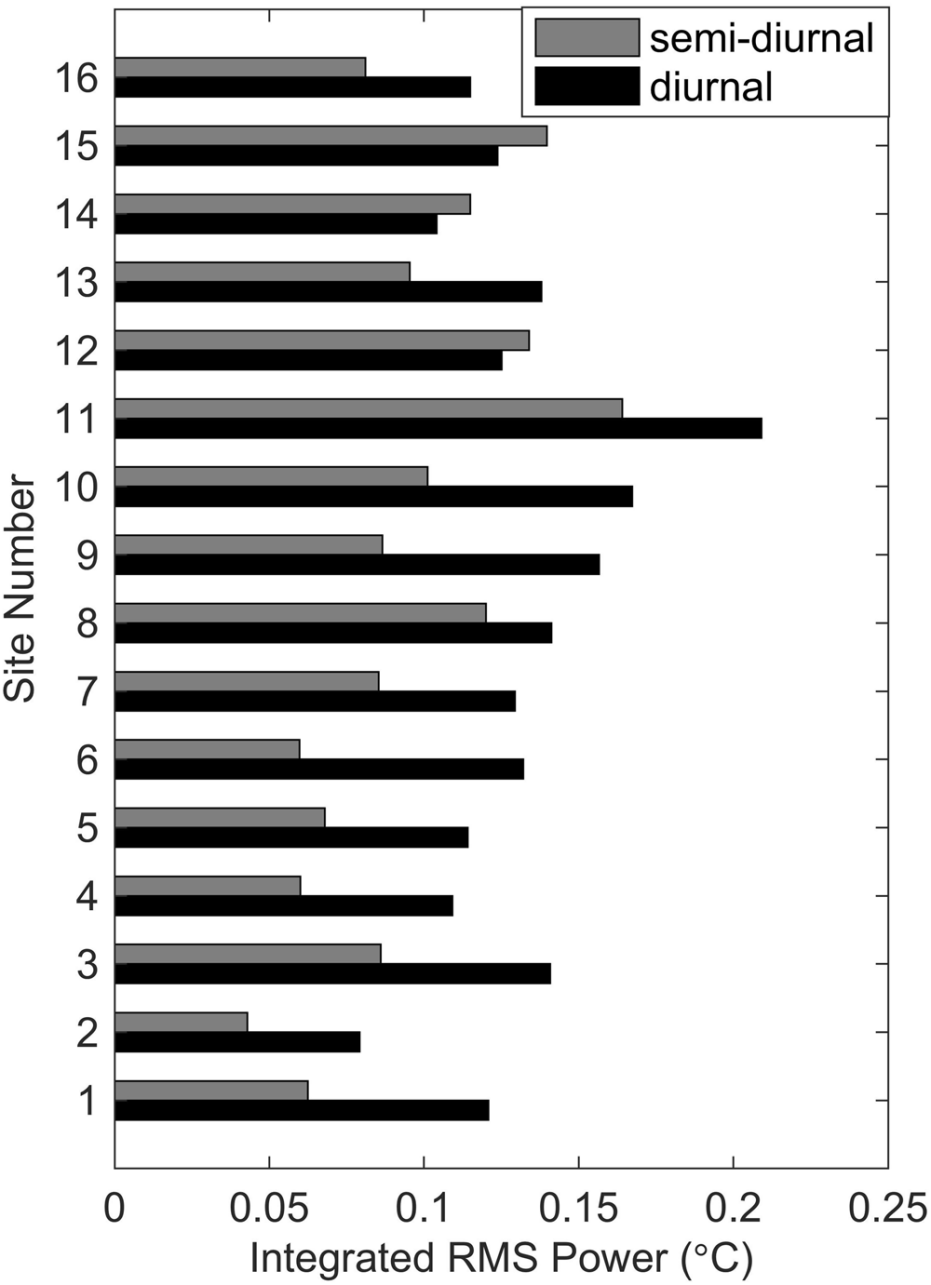
Integrated rms temperature variability in diurnal and semi-diurnal frequency bands across Gulf of California study sites. Diurnal band is defined as 1/33> = *f* >  = 1/20 cycles per hour; semi-diurnal band defined as 1/14 >  = *f* >  = 1/11 cycles per hour based on visual examination of width of prominent peaks in corresponding power spectra shown in Fig. 2.

Along with the power spectra shown in Fig. 2, patterns of the integrated root-mean-square (rms) variability within frequency bands corresponding to the prominent peaks in the power spectra are shown in Fig. 4. The two strong peaks located at frequencies of ~1 cpd and ~1.9 cpd indicate variability concentrated within the diurnal and semi-diurnal bands. Across all but three sites, the variability was greater in the diurnal band than the semi-diurnal. The most variable site, with the greatest variance concentrated in both bands was Site 11 at Isla San Pedro Martir. The three sites where semi-diurnal variability was greater than diurnal are located in the Midriff Islands. For each site in this sub-region, the variability in the diurnal and semi-diurnal bands were comparable. By contrast, sites in the mid and southern portions of the study area exhibited lower overall variability with greater variability in the diurnal than the semi-diurnal band.

### Temperature anomaly return times

Because the temperature variability was primarily associated with rapid (hourly to within-day) cooling events below the mean conditions, we focus our analysis on within-day minimum temperature anomalies. Predicted median return times for daily minimum temperature anomalies of 2–5 °C during the summer period are shown for each site in Table 2. The values are shown for the more variable summer data only, since return times calculated for the low-variability winter observations were mostly very long for all anomalies ≥2 °C, consistent with our other observations that the winter period is characterized by minimal high frequency variability. Table 2 also presents coefficients that can be used to estimate summer return times for cold temperature anomalies of any magnitude and estimated maximum anomaly for any time period using Eq. 2. An important caveat if using the coefficients for that purpose is that return times longer than the 1-yr observational time period or for anomalies larger than actually observed (4 to 5 °C relative to daily running mean temperatures) should be viewed circumspectly, or disregarded.

**Table 2:** Predicted median return times (days) in summer for temperature anomalies (**Δ°)** relative to seasonal running mean conditions for sites across the Gulf of California.

Site #	Site Name	Δ-2°	Δ-3°	Δ-4°	Δ-5°	*α*	*β*	*ε*
16	Puerto Refugio	9	52	936	*	1.02	0.19	0.367
15	Isla Alcatraz	3	6	15	47	1.755	0.201	0.650
14	Punta Quemada	6	14	38	102	0.942	−0.015	0.463
13	Isla Partida	6	19	95	767	1.108	0.139	0.494
12	Isla Las Animas	11	*	*	*	1.013	0.336	0.772
11	Isla San Pedro Martir	2	4	8	21	1.892	0.201	13512
10	Isla San Marcos	5	11	23	42	0.61	−0.216	0.676
9	Isla Santa Inez	5	9	14	22	1.	−0.252	0.165
8	Isla Carmen	6	11	19	30	0.953	−0.245	−0.018
7	Isla Coronado	5	9	15	23	0.610	−0.401	0.368
6	Isla Monserrate	5	11	21	40	1.060	−0.106	0.171
5	Isla Santa Cruz	11	77	*	*	0.913	0.165	0.53
4	Isla San Francisquito	9	27	78	204	0.598	−0.098	0.493
3	El Embudo	6	20	70	269	0.931	0.043	0.471
2	Gallina	*	*	*	*	0.698	0.369	0.243
1	Isla Cerralvo	10	21	38	62	0.441	−0.349	0.223

Coefficient values *α*, *β*, *ε* can be used in Eq. 2 to predict return times for a range of anomaly values. Sites as shown in Table 1 and Fig. 1. * indicates return times >1000 days. Return times in winter (not shown) were all substantially longer than those for summer with most approaching or reaching 1000 days and beyond, with few exceptions.

## Discussion

This study highlights the extensive temporal variability in temperatures on shallow-water rocky reefs along the Baja California Peninsula and the islands throughout the western GOC. Temperatures during the summer and preceding months of seasonal warming were significantly more variable than during the winter and preceding months of seasonal cooling. This pattern is consistent across the GOC and likely reflects seasonal changes in water column stratification and repeated within-day cooling events associated with upwelling forced by winds and internal waves. These data are consistent with the interpretation that in summer and the preceding months of warming surface waters, there is increasing vertical temperature and density stratification in the water column. Three factors likely lead to increased mean temperatures and increased variability in summer than winter: stronger diurnal heating associated with seasonal solar insolation; increased diurnal wind forcing associated with pressure gradients between the GOC basin and surrounding land areas; and increased water column temperature and density stratification that can be expected to support increased internal wave activity at both diurnal and semi-diurnal frequencies^23^. Each of these sources of variability would be expected to result in fluctuations in temperature observed at the fixed depths of the individual temperature sensors and experienced by organisms associated with the shallow water rocky reefs.

The thermal environment at reefs in the Midriff Islands and the central GOC was more variable than at sites to the south. The higher variability during the summer also coincides with the northeastward direction of the average GOC winds^11^, which should promote Ekman transport away from the Baja Peninsula and coastal upwelling along the GOC western boundary^24^. If coastal upwelling and other higher frequency wind-driven processes are contributing significantly to the temperature variability observed during the summer, we would expect variability to diminish beginning in November when the winds begin to subside^10^, followed by a steady decrease in average temperature associated with surface cooling. In fact, we did observe this general pattern at all of our sites (Figs. 2 and 3B). These seasonal differences in temperature variability translate to differences in calculated return times for cold water anomalies at our sites shown in Table 2. While the winter was characterized by limited thermal variability across all sites, the summer was characterized by relatively short return times for cool temperature anomalies in many cases. An analysis of remotely-sensed observations of sea surface temperatures resolved at approximately monthly intervals between 1981 and 2016 across the GOC highlighted latitudinal gradients in mean temperatures and inter-annual patterns associated with ENSO forcing^25^. Our observation of a seasonal pattern of temperature variability on shallow reefs is not unique to the GOC and has, for example, been observed at several sites around the tropical western Atlantic Ocean^26^, at Diego Garcia Atoll in the central Indian Ocean^27^, along southeast Australia^28^, off of central Chile^29^, and in Moorea, French Polynesia^30^.

The higher variability that we observed in the Midriff Islands likely reflects the dynamic oceanography of that region. The Midriffs are recognized as having nearly constant upwelling and strong internal wave activity^15–17^, both mechanisms associated with tidal forcing over a series of shallow sills in that area, driving vertical mixing down to 300 m or deeper^10^. Given the particularly dynamic nature of internal waves and the magnitude of vertical movement of water masses near the Midriff Islands, it is not surprising that temperatures on shallow sites in that region are the most variable that we observe throughout the GOC. Examination of the power spectra support this observation. The pronounced peaks at the diurnal and particularly at semi-diurnal frequency for the Midriff Island sites suggests that a large portion of the total temperature variance may be caused by internal waves in that region. The only three sites where temperature variability was larger in the semi-diurnal frequency band than in the diurnal band are located in the Midriff Islands.

Sites in the central and northern portions of our study area also have the largest annual range in temperature. This results from spatial differences in winter minima across the GOC that are more pronounced than the spatial differences in summer maxima. Similar patterns have also been observed in the western tropical Atlantic Ocean^26^. In both basins, most shallow water sites have maximum temperatures between approximately 30 and 32 °C, regardless of latitude. Minimum temperatures were more variable among sites and regions, and winter minimum temperatures are likely to exert important influences on species ranges especially when low temperatures approach physiological limits for warm water, tropical species.

While the seasonal patterns of temperature variability in the GOC are similar to those in other basins, such as the tropical western Atlantic Ocean, the magnitude of the variability is greater and the likelihood of extreme cooling events is higher in the GOC than described for other regions. For example, the rms temperature amplitude is higher at all of our GOC sites than all of the tropical western Atlantic sites examined using similar methods by Leichter and colleagues^26^. Similarly, although return times for cool temperature anomalies vary across the GOC, many of the GOC sites have shorter calculated summer return times for 5 °C col anomalies than calculated for 2 °C col anomalies in the Florida Keys^31^, one of the most variable parts of the tropical western Atlantic^26^. Clearly, reef organisms at shallow depths (e.g. 5 m) in the GOC are likely to experience highly dynamic thermal conditions.

Among our sites, Isla San Pedro Martir (site 11) exhibited the shortest predicted return times for cold water anomalies (Table 2) and has the largest rms amplitude for the full time series and within the diurnal and semidiurnal bands of the power spectrum (Fig. 4). Isla San Pedro Martir is situated in the east-west center of the GOC and is the southernmost island in the Midriff Islands, therefore likely to be exposed to dynamic oceanography as a result of its proximity to both the GOC’s eastern and western boundaries and to the active areas in the Midriffs.

Although we focus here on physical patterns of temperature variability and minimum temperature anomalies, there are significant potential biological and ecological implications of the physical variability we measured across the study region. The magnitude of temperature variability in the GOC is high, and individuals of species with geographic ranges that cover several of our sites are likely to experience a range of thermal regimes across latitudes. In some cases, these species have single populations with geographic ranges that cover sites with widely different temperature variability while others show distinct population structure that parallel to some extent these oceanographic patterns^32,33^. These differences are likely even greater for populations with wider distributions that extend far south, or north of the GOC. Generally, changing ocean temperatures have been shown to facilitate and drive shifts in species distribution, both geographically^8,9^ and across depths^34^.

Marine species experience physiological limits associated with both minimum and maximum temperature thresholds, and these thresholds can change on evolutionary timescales, with some pairs of sister species exhibiting tolerance to substantially different temperature ranges^2^. In some systems, temperature variability is implicated in changes to marine fish behavior^3^ and success in colonization of new areas^7^. In the northern GOC, temperature variability is known to affect the interactions between species with relatively narrow distributions and those with relatively wider distributions^4^. In that case, the most extreme thermal events favor the species with narrower distributions, potentially a result of individuals of those species being more tolerant of a variable environment. Even though species with broader distributions experience larger temperature ranges across their entire geographic ranges, individuals may be unable to tolerate the extreme variability at a single site such as that of the northern GOC. Given the mosaic of environmental conditions we observed, successful GOC species are likely to be those that are also highly tolerant of temperature variability. Quantifying fine-scale spatial and temporal temperature variability can provide an important tool for studying reef faunas in this and other regions. Wider incorporation of detailed data on environmental variability promises increased insights into our understanding of the factors controlling the fine-scale distribution, abundance, movement and ecology of marine species.

## Methods

### Observations

As part of a large-scale study to characterize GOC rocky reefs, we collected *in situ* temperature data from 2009 to 2011. Onset Computers Hobo Pro v2 submersible temperature data loggers (0.2° accuracy, 0.02° resolution, 5 min response time) were deployed on 16 reef sites from Puerto Refugio at the northern tip of Angel de la Guarda Island in the Midriff Islands to Cerralvo Island south of La Paz Bay (Fig. 1). Sites were chosen based on previous faunal surveys and ongoing semi-regular monitoring of fish communities across the GOC. At each site, two loggers were installed directly to the rocky reef surface using stainless steel eyebolts attached with marine epoxy. To mitigate against loss or failure of individual loggers, redundant paired loggers were positioned within one meter of each other at the same depth at each site. Installation depth was typically 5–6 m, but reef bathymetry and proximity to survey areas required some loggers to be installed slightly shallower. The data loggers recorded temperature at 20-min intervals from July 2009 to July 2010 at most sites and through May 2011for some sites. For each site where two data loggers were recovered, the resulting time series were very similar or identical at most time points. We used the mean value from the two loggers at each time point, or in cases where only one logger was recovered, the resulting single time series was used.

### Analyses

#### Descriptive statistics

We determined the daily minimum, maximum, mean, and standard deviation for each site over the full deployment period. To examine seasonal patterns, we calculated 29-day centered moving averages for each site and also considered the high- and low-frequency thermal variation for the Summer (May-October) and Winter (November-April) seasons, separately.

#### Time series analysis

To characterize the magnitudes and frequencies of temperature variability at each sampling site, we calculated power spectra for each site and season using a Fourier transformation (FFT) and Welch’s method of averaging replicate spectra calculated from 14-day (1008-data point) sections of the data with an applied Hamming window and 50% overlap among data subsections^35^. For each site we then estimated the root mean squared (rms) area within two frequency bands corresponding to diurnal and semi-diurnal variability. We defined ranges of the these bands following visual inspection of the frequencies containing the two prominent peaks in the power spectra across sites, with diurnal as 1/20 to 1/33 cycles per hour – corresponding to periods between 20 and 33 hr, and semi-diurnal as 1/11 to 1/14 cycles per hour – corresponding to periods between 11 and 14 hr. Because the estimated magnitude of the integrated power in each band is sensitive to the choice of band widths, these were held constant for the analyses across the study sites.

#### Minimum temperature anomalies and return times

For each day in each temperature record, we calculated the minimum temperature anomaly as the difference between that day’s minimum temperature and the 29-day centered moving average. These values were then used to calculate the estimated median return time in days for a given cooling event as well as the estimated magnitude of the most extreme cooling event that would be expected over a given time period^36–38^. These methods for estimating return times have been applied to ecological data^39,40^ and to a temperature time series^31^ and follow a four step process to determine the probability that an extreme value, *x*_*i*_, in a single time interval will be less than or equal to a given value, *x*:

1 $$P(x)={\rm{Prob}}({x}_{i}\le x)$$

To estimate the probability function, *P*(*x*), we: (1) divided the data into a series of equal length intervals (1 day); (2) recorded the extreme value (the largest positive difference between the running mean temperature and the daily minimum temperature) in each interval; (3) ranked extreme values by magnitude; and (4) fit a continuous probability function to their cumulative distribution^41^. A generalized extreme value equation can be used for fitting a continuous probability distribution to a set of extreme values. In this case the extreme cool temperature anomalies selected from each sampling day represent the tail of the distribution of all of the observed temperature anomalies. With the underlying 20 min sampling interval of the temperature time series, there are 72 points per day and this method selects the only the largest of those values in each day and then fits a continuous function to the distribution of those extreme values. Under assumptions that the anomaly measurements are independent and stationary through in time, this probability function has been shown to approach an asymptotic form^37^:

2 $$P(x)=\exp -{[(a-\beta x)/(a-\beta \varepsilon )]}^{1/\beta }$$

with the following qualifications:

if *β* > 0, *P* = 1 for *x* ≥ *α*/*β*

if *β* < 0, *P* = 0 for *x* ≤ *α*/*β*

Following prior application of this technique^31^, in order to better satisfy the assumption of independence of the daily minimum temperature anomalies, prior to fitting Eq. 2 we sub-sampled the anomaly time series taking the values for every fourth day, an interval beyond which the time series serial auto-correlation was non-signficiant. In considering the assumption of stationarity of variance through time (which is clearly not the case for many environmental time series), we divided the time series and calculated the cooling anomalies separately for the more-variable summer and the less-variable winter periods. Recognizing these assumptions we also note as a caveat and disregard any return time estimates for anomalies of larger magnitude than those actually observed and also for times that are longer than the observed 1-yr time series (see Results). Estimates of *α* (the rate of increase of *P*(*x*) with the natural logarithm of time), *β* (which, when divided into *α*, estimates the maximum achievable extreme value), and *ε* (the mode value) were found using maximum likelihood, nonlinear curve fitting in Matlab and used to solve *P*(*x*). The estimated return time, *τ*(*x*), represents the predicted median number of days between successive extreme values as large as *x*, and is given by the inverse of 1 − *P*(*x*):

3 $$\tau (x)=1/(1-P(x))$$

Because our inspection of the high- and low-frequency time series (Fig. 2) showed the temperature changes were mainly associated with rapid, within-day cooling events below the running mean temperatures, we determined it was not useful to repeat the calculations for warm temperature anomalies with the available data. We surmise that a useful analysis of warming anomalies, that could consider for example anomalies associated with oceanographic and atmospheric extreme heating events would likely require significantly longer time series capturing inter-annual variability for example associated with ENSO.

## Data Availability

All raw data will be made freely available by the corresponding author upon reasonable request. Data and supporting analytical methods and programs are available at https://github.com/grantlygalland/temp-return-times.
